# Thermally induced collision of droplets in an immiscible outer fluid

**DOI:** 10.1038/srep09531

**Published:** 2015-05-07

**Authors:** Ashkan Davanlou, Ranganathan Kumar

**Affiliations:** 1 Mechanical & Aerospace Engineering, University of Central Florida, Orlando, Florida 32816, USA

## Abstract

Micro-total analysis systems (μTAS) have attracted wide attention and are identified as a promising solution for sample transport, filtration, chemical reactions, separation and detection. Despite their popularity, the selection of an appropriate mechanism for droplet transport and coalescence has always been a challenge. This paper investigates the use of Marangoni flow as a mechanism for levitating and transporting droplets on immiscible liquid films at higher speeds than is possible currently. For the first time, we show that it is possible to realize the natural coalescence of droplets through Marangoni effect without any external stimulation, and deliver the coalesced droplet to a certain destination through the use of surface tension gradients. The effects of shape and size on collision outcome are studied. Regions of coalescence and stretching separation of colliding droplets are delineated based on Weber number and impact number. In addition, the effect of viscosity on post collision regimes is studied. The findings in this fundamental study can be beneficial to many applications such as welding, drug delivery and microfluidics devices in controlling small droplets and targeting them to various locations.

The emergence and advancement of digital microfluidics have facilitated the design of novel systems that can perform fluidic operations^1^,^2^. A variety of methods of droplet actuation have been developed including Marangoni convection, dielectrophoresis, electrowetting-on-dielectric (EWOD), acoustic-driven and optoelectrowetting. Among these, Marangoni convection is a promising technique that has gained wide attention for droplet-based microfluidic system. Surface tension gradients on very thin liquid films can set up motion due to unbalanced forces at the interface. This is called the Marangoni effect. The possibility of manipulating droplets in an open structure, contact-free, without droplet pinning and surface contamination are some of major advantages of thermocapillary actuation^3^,^4^. In several applications, drop-drop interactions play a central role in the performance of the system^5^. For instance, separation and analysis of components of a mixture, or transport and mixing of chemical reagents to form new compounds are critical to drug delivery. Recently, Javadi et al.^6^ showed the feasibility of fabrication of a bidirectional capillary micropump by engineering the surface properties through electrowetting. So far, droplet interactions in air and collision of droplets on solid surfaces have been studied vastly in the literature. The collision behavior of liquid droplets floating on liquid substrates opens up interesting possibilities and as yet, remains unexplored.

The coalescence of liquid droplets is ubiquitous in nature and is of great interest to wide range of processes, such as rainfall^7^,^8^,^9^,^10^, droplet combustion^11^, ink-jet printing^12^, microfluidics^13^ and separation of emulsions^14^. Collision of binary droplets in air is a well-established phenomenon that is studied in depth by different authors^15^,^16^,^17^,^18^,^19^,^20^. In a benchmark paper, Qian and Law^18^ reported five distinct regimes of drop-drop interactions for hydrocarbon and water, namely (I) coalescence after minor deformation, (II) bouncing, (III) coalescence after substantial deformation, (IV) coalescence followed by separation for near head-on collisions, (V) coalescence followed by separation for off-center collisions. Based on their results, the droplet velocity, fluid properties and impact parameter were found to be the determining factors in the collision outcome^16^,^18^. In addition, coalescence of sessile droplets on a substrate (sometimes mentioned as confined droplet collision) was the focus of few recent studies, in which the coalescence is realized by condensation, addition of volume to one droplet or using pressurized air^21^,^22^,^23^,^24^. The focus of these works was the dynamics of meniscus bridge upon early stages of coalescence^23^,^25^,^26^, contact angle hysteresis^24^ and timescales^27^. Recently, Thoroddsen et al.^28^ and Kavehpour^29^ reviewed the coalescence process of liquid drops on thin liquid layers and dry surfaces. Also wetting and spreading of fluids on solid surfaces discussed using contact angle, surface heterogeneity and contact lines^30^,^31^.

In this paper, for the first time, we investigate the coalescence phenomenon of two levitated droplets. When a droplet is released from a certain height (typically 1.5–4 times its diameter), it can overcome the impact, preserve its spherical shape, and stay levitated on the surface^32^,^33^. This can be seen in Fig. S1 of Non-dimensional release height versus Bond number (Bo) for several droplet diameters in the range of 1.4 mm to 3.9 mm. This gives rise to Bo in the range of ~2 to ~10, i.e., buoyancy force changes from a similar magnitude of surface tension force to an order of magnitude higher. The droplet at higher Bo may not be levitated if it is not released from a height very close to 1.5 times. The levitated droplet is not in direct contact with the free liquid surface due to the existence of an air gap of a few microns thick between two media^34^,^35^,^36^. A submerged droplet, also called a sessile droplet, on the other hand, is partially engulfed in the carrier fluid and has a lens-shape configuration. In order to trigger the movement of droplets, a thermal gradient on the surface is created by passing current through embedded heaters. This leads to a surface tension gradient, and deformation at the liquid/air interface thus creating motion of droplets towards the heating source^3^. Although at first this might seem counter-intuitive, the surface slope due to gravity counteracts the Marangoni force inside the liquid film, consequently the droplets migrate towards the heater. In the case of submerged sessile drops, they behave like tracers and their movement is in the direction of Marangoni flow, away to the heating source. Understanding the physics of droplet collision on liquid films is beneficial, as many biochemical reactions take place in such environments rather than solid substrates. In addition, the droplet transport is faster and it would be possible to handle the biochemical reagents in a simple but practical manner. There has been no study of natural or passive coalescence of droplets on a thin film.

We address the collision of droplets levitated on Marangoni flow of a second immiscible liquid. Non-dimensional numbers such as Weber number and impact number are used to explain the phenomenon. Later, we used a combination of different fluids to study the effect of viscosity on coalescence. The results in this work reveal that by creating a thermal map, it is possible to transport droplets to desired locations precisely, and also to drive droplets naturally to coalescence and mix them. The current work provides an opportunity for better understanding of thermo-coalescence phenomenon and an optimal design of droplet-based systems.

## Results

**Figure 1:**
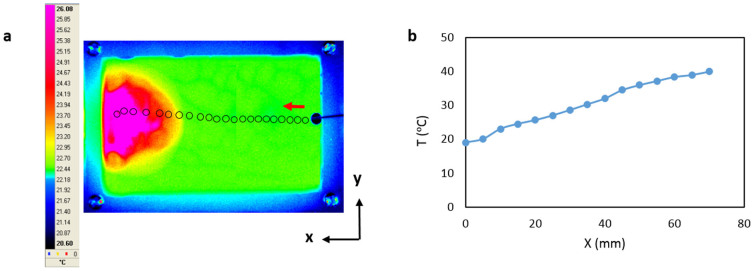
(a) The trajectory of a 3 mm droplet for a temperature drop, ΔT, of 20°C across the channel is shown on an infrared image. The black circles represent the position of the droplet at every 14 frames; (b) Liquid film's temperature profile along the y-axis symmetry line of the channel from IR measurements. The heater is turned on right after the droplet is released.

First we study the motion of a levitated droplet on a liquid surface due to thermocapillary effect. Due to the applied temperature gradient inside the liquid film Marangoni flows will form that circulate the carrier liquid. This phenomenon is accompanied with liquid surface deformation. As a result the droplet migrates towards the hot side of platform. Figure 1a shows the droplet trajectory as a result of an applied 20°C temperature difference between both sides of the platform. In order to create a sharp surface gradient, an electric pulse is given to the embedded resistor. In Fig. 1b the temperature profile of the carrier liquid along the symmetry line of the platform is plotted. The data are acquired from the infrared camera.

**Figure 2:**
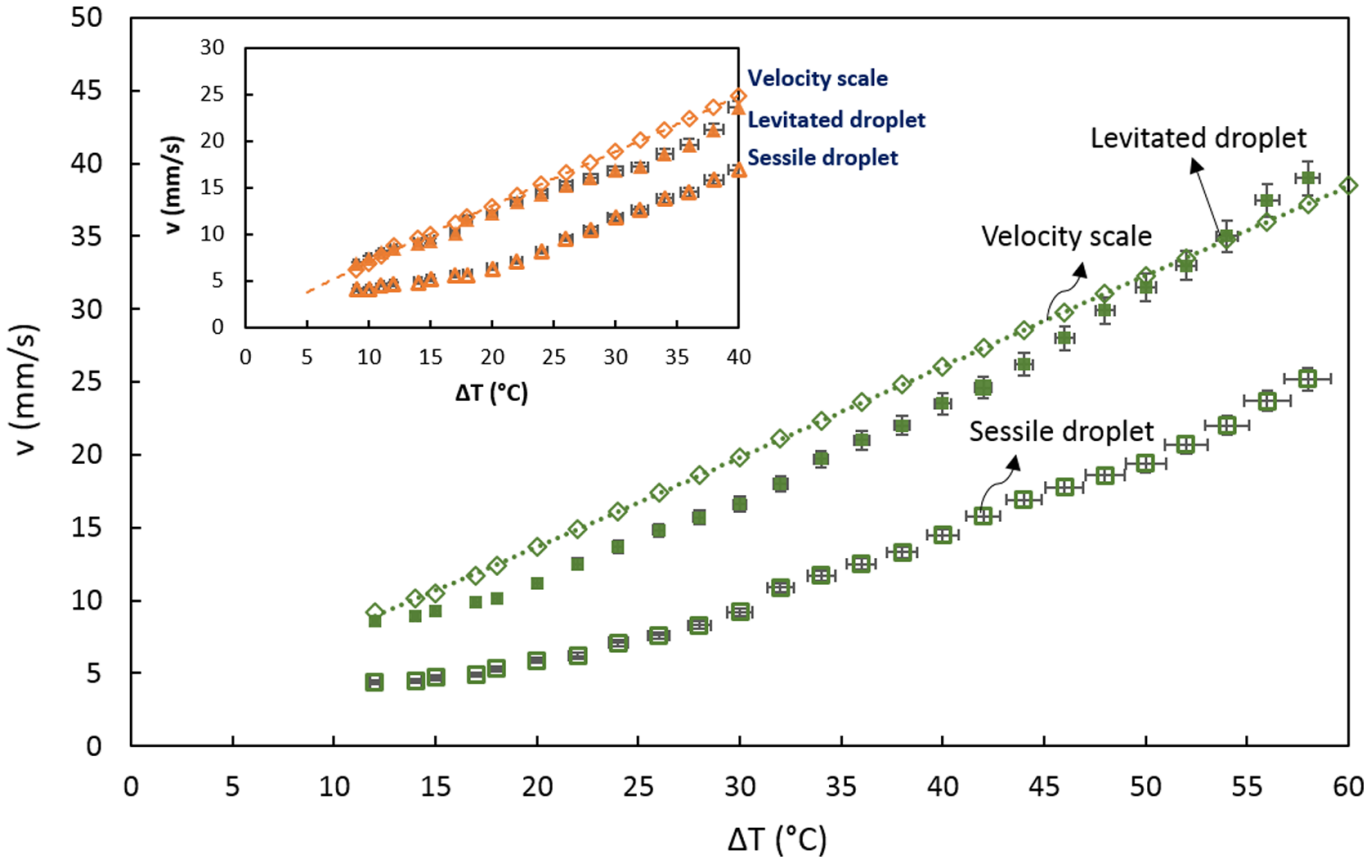
Variation of droplet velocity with temperature difference (ΔT = 60°C). Velocity scale (theoretical), sessile drop velocity and spherical drop velocity (experimental) for a 3 mm water drop are shown. The velocity scale data are curve-fitted to show the trend. The inset shows experimental results compared to velocity scale for ΔT = 40°C.

Figure 2 shows the effect of an imposed thermal gradient along the channel on droplet velocity. Droplet velocity is measured by analyzing the trajectory of droplet motion while it migrates towards the heater using IR camera images, and it is calculated by measuring the distance a droplet migrated after 12 consecutive frames. The temperature difference is measured for the new droplet position at the beginning of each 12 frame interval with respect to the drop dispensing point. The velocity values are cross-validated using high speed imaging for the same motion. The calculated uncertainty of the measurement was ~4% for velocity and ~2% for ΔT. The procedure is repeated for ΔT = 40°C. Average velocities of the levitated droplets and sessile droplets which move in opposite directions are plotted against the velocity scale, given by
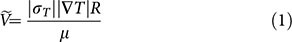
where μ is the dynamic viscosity, R is the droplet radius, σ_T_ is the rate of change of interfacial tension with temperature, and ∇T, the temperature gradient imposed on the thin film^37^. Typically, spherical-shape droplets gain higher drift velocities in magnitude, although they migrate against the induced Marangoni flow. The lens-shaped droplets act similar to tracer particles. The size effect is favorable for spherical-shaped drops as they can reach higher drift velocities for the same temperature difference (ΔT), with the sessile droplets displaying the opposite effect due to larger drag for larger droplets (not shown here). Although all cases follow these trends reasonably well, the sessile drop velocities are significantly lower compared to the velocity scale, while the levitated droplet have similar values.

**Figure 3:**
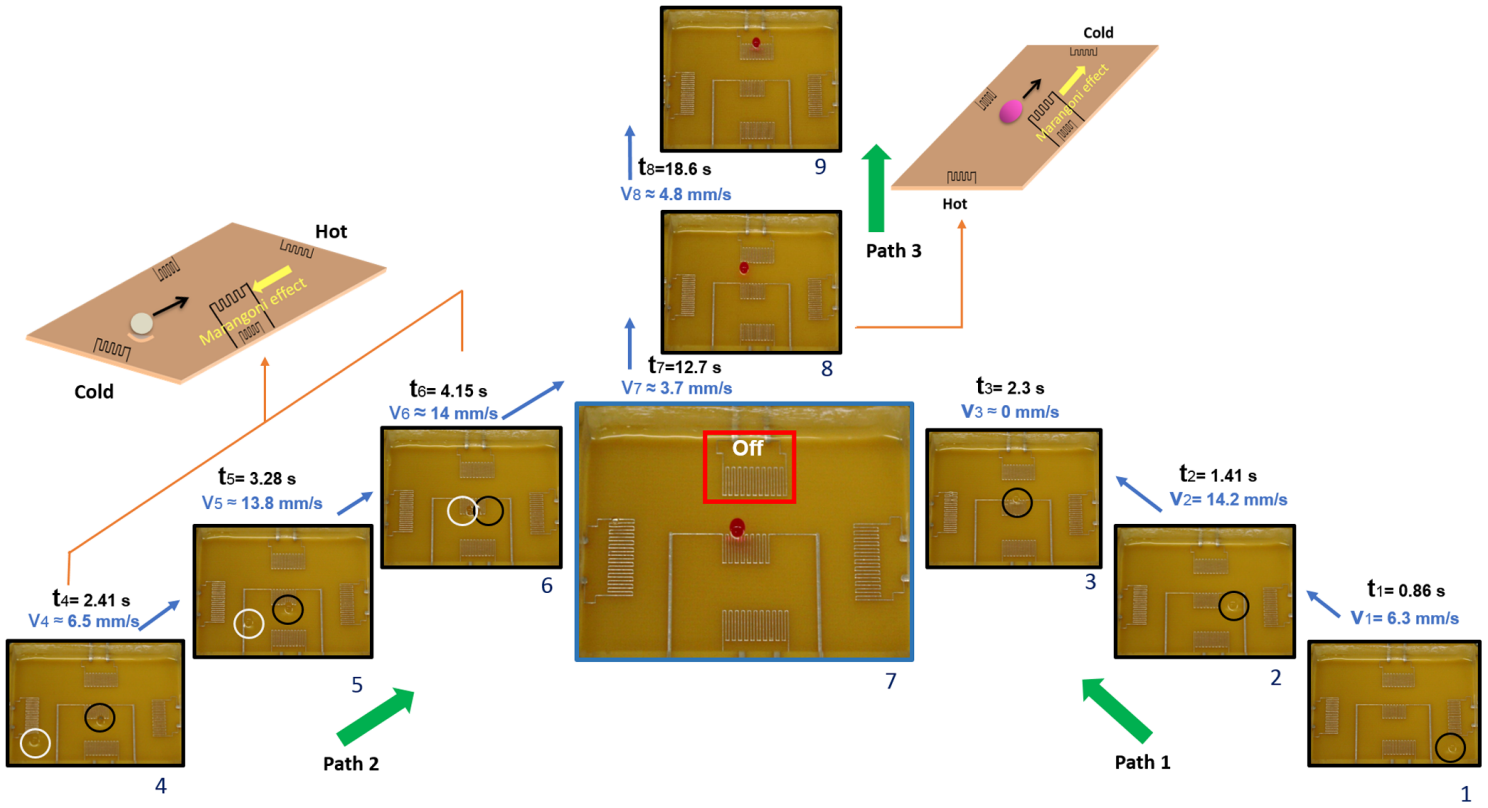
Transport and coalescence of levitated droplets. Initially, all heaters except the one shown as ‘off’ are active. The first droplet of potassium hydroxide is levitated at one end of the printed circuit board, and is allowed to flow through Path 1. After it migrates to the more powerful central heater, a second levitated droplet, phenolphthalein, is allowed to flow through Path 2 under the same condition. The drops collide and mix into a larger pink droplet. With continuous heating, the air layer underneath the droplet is ruptured, and the droplet sinks and becomes a sessile drop without being pinned. The sessile drop then moves towards lower temperature through Path 3. This dual migration of droplet moving towards or away from the heat source is governed by the thermocapillary effect due to the drop shape effect. The inset on the left shows the schematic for the migratory behavior of the levitated droplet against Marangoni convection. The inset on the right shows the schematic for the larger coalesced sessile droplet moving away from the heat source in the direction of the maximum temperature gradient.

The droplets are released from a constant height (~1.5 times their diameter) on a thin film of Fluorinert oil so that the droplets can levitate. The collision of droplets is realized through thermocapillary effect, i.e. surface tension gradient which is caused by thermal gradient at the interface. Fig. 3 shows the transport of two equal-sized droplets to the center of the platform at different times along different paths. When the central heater is turned on, surface deformation occurs that allows the levitated droplet to move against the Marangoni flow underneath, floating towards the heater where the deformation is maximum (Frames 1–3). Once the droplet reaches the heater, the heater functions as a thermal trap, and the droplet remains stagnant at the center part of the platform (Frame 3). When the second droplet is released (Frames 4–6), this droplet also moves to the central heater where the surface depression is maximum, head-on collision (b ≈ 0) occurs at the central heater in Frame 7, since it has higher power compared to the other embedded heaters. In order to illustrate the mixing phenomenon, the first droplet dispensed is potassium hydroxide and the second one phenolphthalein which is used as a pH-indicator. Both droplets are initially colorless, but when phenolphthalein is in contact with basic solutions (pH > 8.2), it turns pink. After 5.01 s, the two droplets coalesce, mix and form a larger levitated droplet that has a pink color. This large drop is at first thermally trapped at the heater. However, when the droplet temperature reaches the liquid film temperature, the spherical droplet ruptures the air layer underneath, submerges in the carrier liquid, and transforms to a more stable lens-shape sessile configuration^32^,^34^.

Since all heaters are active from the beginning of the experiment except the one on top (as indicated by ‘off’ in Frame 7), a local cold spot is created in that region, and as pointed out earlier, a lens-shape drop follows Path 3 and migrates to the colder region that has higher surface tension forces. The sessile drop moves preferentially to the top instead to one of the other heaters (at low intensity) because of the maximum temperature gradient in this direction. Now, the question is whether the collisions occurring on a thin film will have similar outcome as collisions under free flying condition. In the next section, we will investigate droplet migration due to Marangoni effect and the possible scenarios when impact parameter and Weber number change.

**Figure 4:**
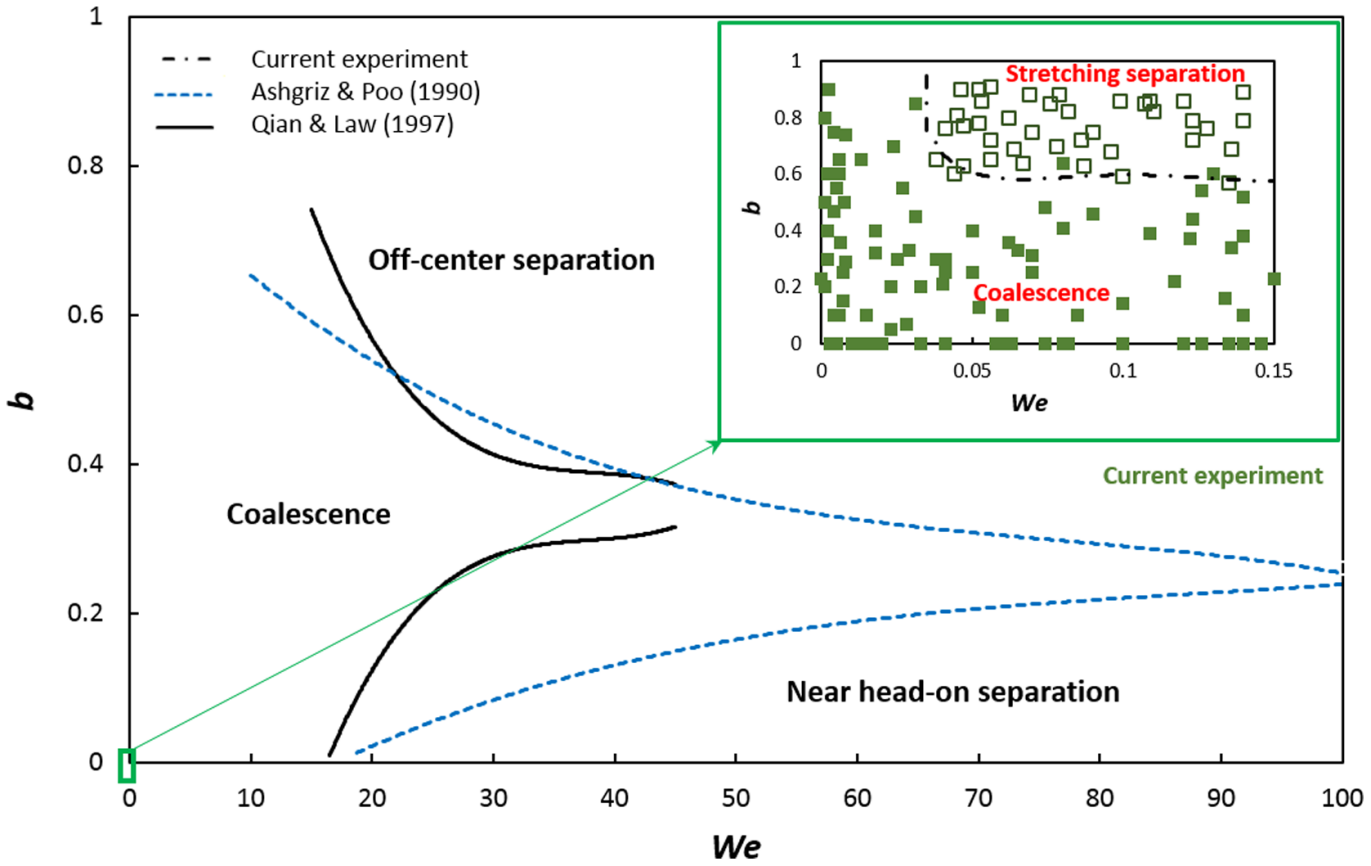
Analytically obtained regions of coalescence, reflexive separation, and stretching separation for equal-sized water droplets based on Refs. 15 & 18. The inset shows that collision of drops on a thin film leads to two distinct regimes at very low Weber numbers (2.5 mm ≤ D ≤ 3.6 mm). Each square shows one experiment.

Weber number (We) is the ratio of the inertial force to the surface tension force, and the impact parameter (b) represents the relative distance between the centers of the two droplets. These non-dimensional parameters are typically used to explain the collision process of the droplets. For equal-size droplets, We and b are defined as 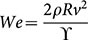 and *B/2R*, respectively, where ρ is the droplet density, ϒ is the interfacial tension between the droplet and thin film, *v* is the relative velocity and *B* is the projected distance between the droplet centers in the direction normal to vector *v*. We will now investigate the post collision regimes of a water droplet as impact parameter and droplet velocity vary. Velocities are low in Marangoni flow, and the range of Weber numbers reported here is two orders of magnitude smaller than for unconfined forced droplet collisions in air. Figure 4 shows the reported interaction of water droplets in air classified into three regimes, while the inset depicts the observed regimes in the current study.

At low impact velocities, i.e., low We, the collision of droplets results in an immediate coalescence even if occurs at high impact parameters. At higher impact velocities, beyond We ~0.04, the impact parameter determines the outcome of the collision. For b > 0.6, stretching separation rather than coalescence could be the dominant regime. The results include the collisions of a stationary droplet with an incoming droplet as well as two moving droplets.

**Figure 5:**
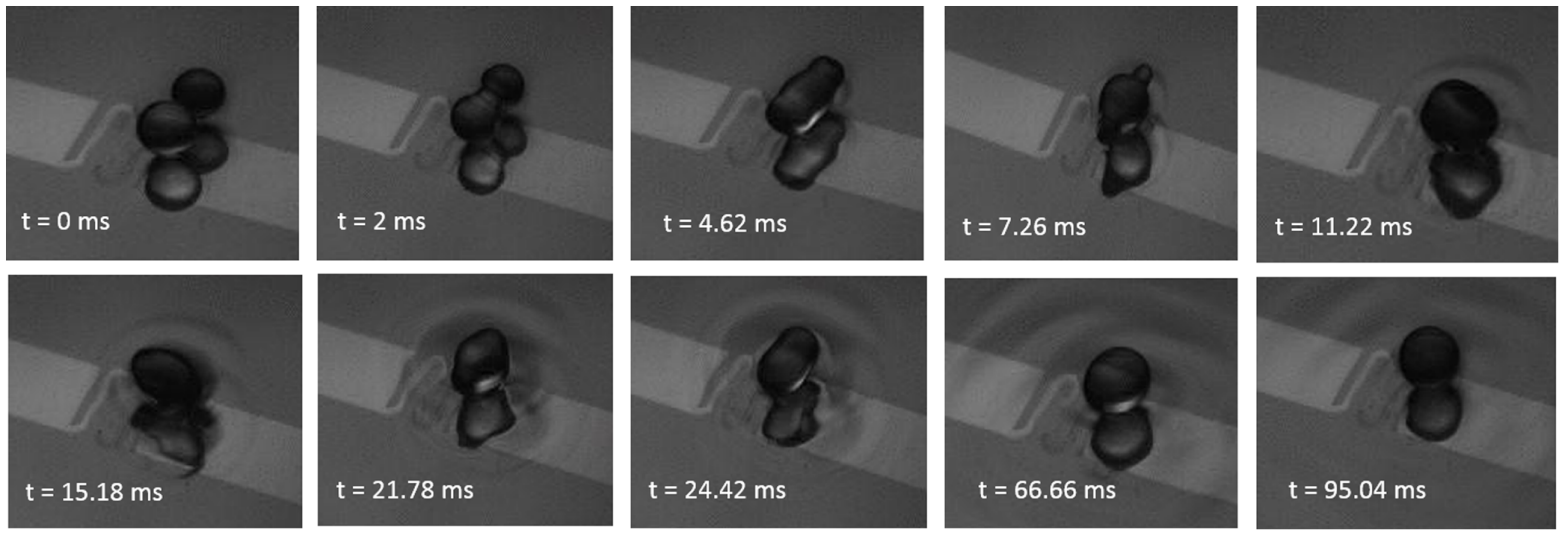
A sequence of high-speed images show the stages of head-on collision that lead to permanent coalescence (ΔT = 20°C, D = 3 mm, We = 0.012). Both the droplets and their reflection are seen until they coalesce.

At low impact velocities in the coalescence regime, the two droplets form a single drop and subsequently mix as seen in Figure 3 using the pH indicator. The coalescence phenomenon advances in three successive stages: 1) approach and collision of drops from an initially large separation, 2) film drainage that occurs when the separation distance is asymptotically small compared to the drop radii, 3) film rupture as a result of instabilities. The coalescence can only occur if the air film thickness reaches the critical value of 10^2^ A° or less^26^. In this case, they form a larger droplet with a mass equal to sum of the mass of primary droplets. Yi et al.^38^ reported that the rise in droplet temperature increases the probability of coalescence after the collision of droplets. Figure 5 shows the coalescence of 3 mm levitated droplets at a camera speed of 2000 fps, where different collision stages are noticeable. As the drops collide, they attain a dumbbell shape initially. When the ambient fluid between them is pushed away, they first become tubular and later into a stable larger spherical droplet.

Reflexive separation is known to occur for near head-on collisions, while stretching separation (also called off-center separation) occurs at moderate to large impact parameters^15^. In stretching separation, after droplets collide temporarily to a combined mass form and eventually separate into two or more drops. This is attributed to the competition between the kinetic energy of the untouched part of the drop and the surface energy of the interacting part^15^,^39^. Stretching separation may follow by fragmentation in which satellite drops form.

**Figure 6:**
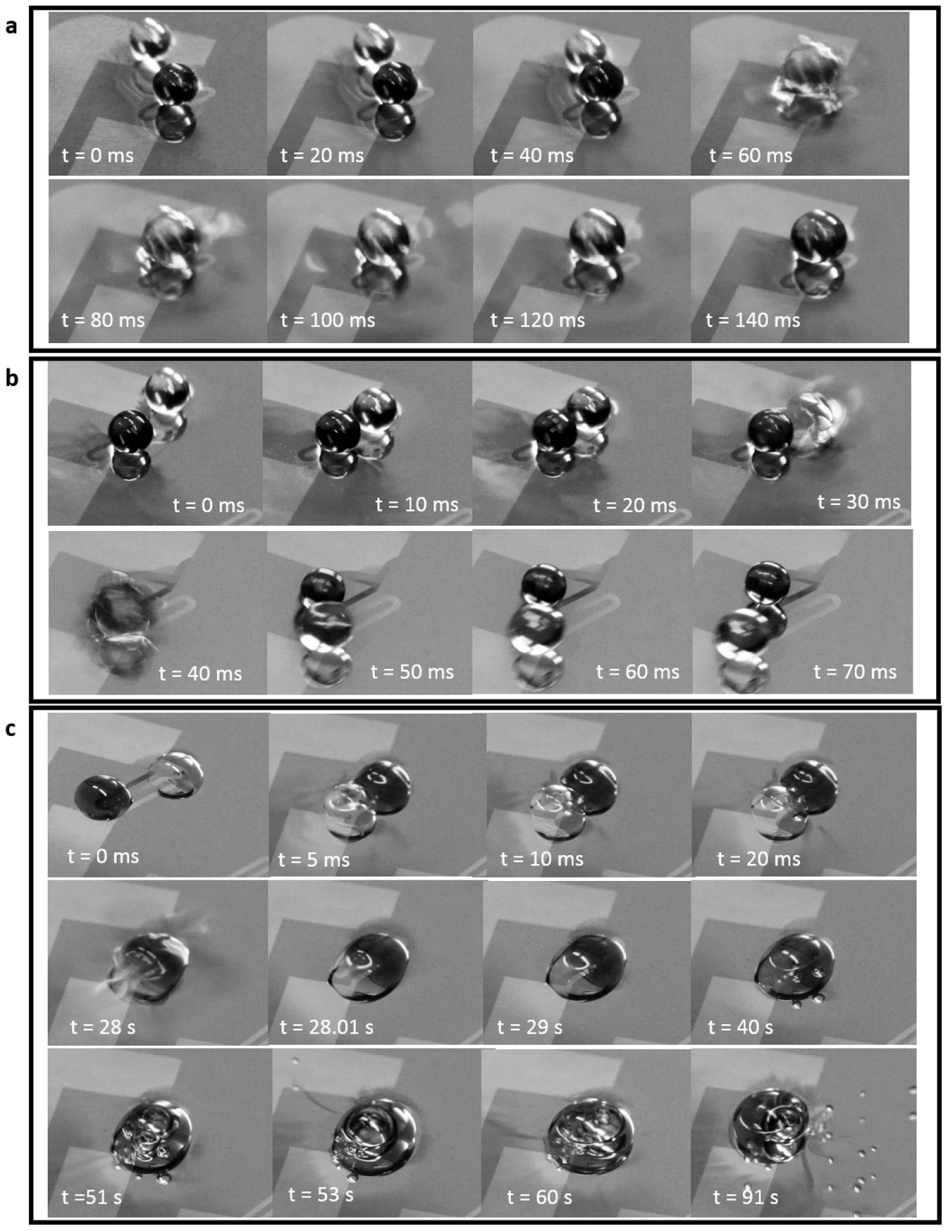
Snapshots of the collision of identical droplets for ΔT = 20°C. (a) Coalescence of spherical-shaped water droplets (D = 3 mm, v = 10.2 mm/s, We = 0.012); (b) stretching separation of spherical-shaped water droplets (D = 3 mm, v = 10.2 mm/s, We = 0.012); (c) coalescence of sessile water droplets on a liquid substrate (D = 2.5 mm, v = 14 mm/s, We = 0.0067).

The two major modes that are observed in collision of levitated drops are shown in Figures 6a and 6b. In these snapshots one droplet is colored with blue dye to better show the interactions. Coalescence is facilitated by surface tension forces at low impact velocities (Fig. 6a). At low impact parameters, the relatively high kinetic energy (higher ΔT′s) helps the impacting droplet to break the air film faster compared to lower ΔT′s. For stretching separation, there is no obvious formation of liquid bridge between the droplets^38^. After collision, a minor deformation occurs and each droplet is seen to continue in its own path while it is still levitated (Fig. 6b).

The coalescence of the sessile droplet is independent of their impact parameter. Such droplets that are partially submerged may convert to pinned droplets if either the thickness of liquid film below them shrinks significantly due to a large imposed thermal gradient or the size of the formed droplet is so large that it touches the solid substrate. In Figure 6c, the coalesced droplet gets pinned, evaporates after a few seconds and undergoes a phase transition. The rise of the droplet temperature will increase the vapor pressure. The accumulation of vapor around the droplet helps push out air at the interface upon impact, therefore the possibility of coalescence after collision increases^18^,^38^. In this study, we used liquids such as silicon oil and water at atmospheric pressure, which have relatively low vapor pressure. It is also important to mention that the process of coalescence of levitated droplets typically occurs in milliseconds (Figure 6a). The low thermal conductivity of the carrier liquid (0.066 W/m°C) helps avoid an excess temperature rise in floating drops. Therefore, the vapor pressure effect was seen to be minimal for the pair of fluids used in our study. Compared to the coalescence of levitated drops, the coalescence time for sessile droplets is three orders of magnitude greater. This is comparable to reported values in the literature^40^,^41^ for sessile drops on super hydrophobic surfaces. However, due to lower impacting velocities, in the current experiments, other types of interaction such as the rebound or disruption of the coalesced droplets were not observed.

## Discussion

**Figure 7:**
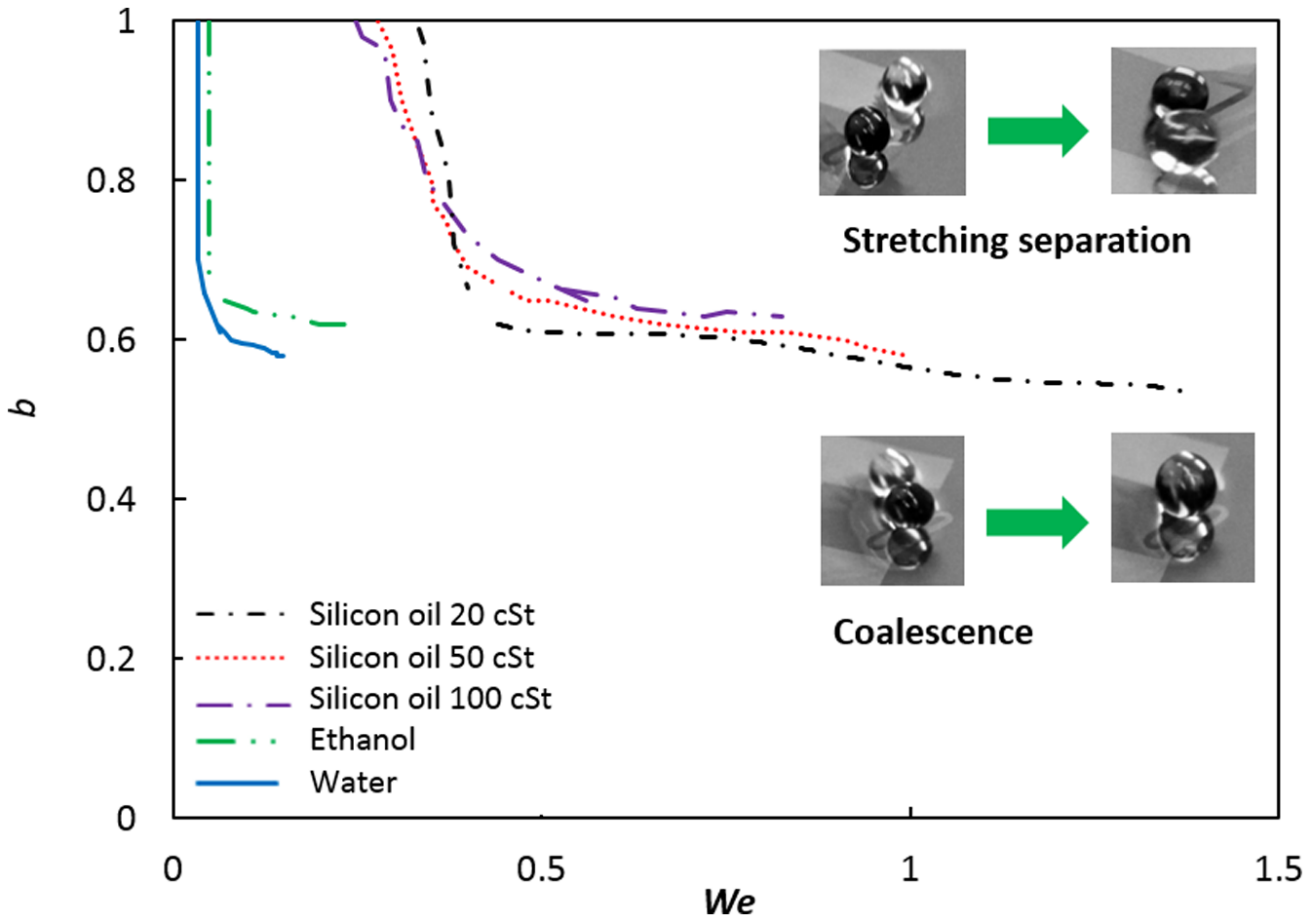
Impact number vs. Weber number for silicon oil drops (D = 3 mm) of various viscosities. Increase in viscosity leads to higher probability of coalescence even at slightly larger impact numbers.

Viscosity has a major effect on the transport velocity of the levitated droplets and hence coalescence. Here, the thermocapillary effect causes surface deformation of the liquid film, however a lower viscosity fluid can reach higher velocities. For the same ΔT, when the viscosity is larger, the collision kinetic energy (CKE) is lower due to low velocities. In general, when the droplets coalesce, the coalesced droplet has a smaller surface area than the sum of two pre-collision droplets^15^, thus there is a decrease in surface energy from pre- to post-collision. In this case, the surface energy stays the same since the original dropsize is the same. However, CKE reduces due to higher viscosity, promoting coalescence (Figure 7). In addition, earlier coalescence occurs since both CKE and final surface energy (which is the difference between pre- to post-collision surface energy) are dissipated through work done during the breakage of the air film^16^. In Figure 7, the coalescence process is facilitated easier at higher viscosities, thus shifting the transition line upwards, indicating that the impact parameter i.e., the separation distance, can be higher for higher viscosity fluids to merely graze and coalesce.

In summary, our recent discovery^3^ that levitated droplets float in the opposite direction of surface velocity of a liquid layer undergoing Marangoni convection is exploited to naturally coalesce droplets on liquid substrates. The collision behavior of identical drops is studied experimentally as a result of a thermal gradient on a thin film. The existence of the transition line between coalescence and stretching separation in this passive mode of transport is similar to what was observed in the literature for force coalescence at significantly higher Weber numbers^15^,^16^,^17^,^18^. This study offers new insight to thermo-coalescence and demonstrates how natural coalescence could be used to transport, mix and collect reagents in an efficient manner. The results can be employed to enhance performance of self-cleaning surfaces, thermal diodes and micro-total analysis systems. As future work, the role of Marangoni effect on droplet mixing will be studied.

## Methods

### Device fabrication

A double-sided 96 × 64 mm^2^ board is used as the main platform. The laminate is 1.57 mm FR-4 epoxy glass with 36 g of copper. Pads are plated with a tin/lead solder. The solder mask layers are green LPI and the etching resolution is 0.15 mm. A custom made acrylic sheet is used to create a sealed container for liquid film on top of the printed circuit board (PCB). The sheet is mounted on the board using screws and adhesive sealant. The wires are back soldered to the board. As the board does not have the typical green solder mask, the yellowish color of FR-4 laminate is dominant.

### Material used

Fluorinert FC-43 (SynQuest Laboratories, Cat. No. 3132-2-43) is used as carrier liquid for droplet transport. FC-43, is a chemically inert and thermally stable liquid. The thickness of the liquid film is 2 mm at room temperature. Droplets are made of Potassium Hydroxide (LabChem, Cat. No. LC19275-1) and Phenolphthalein (Ricca Chemical Co., Cat. No. 5600-16). The properties are summarized in Table S1. The platform is cleaned with acetone and methanol between experiments.

### Experimental setup and method

**Figure 8:**
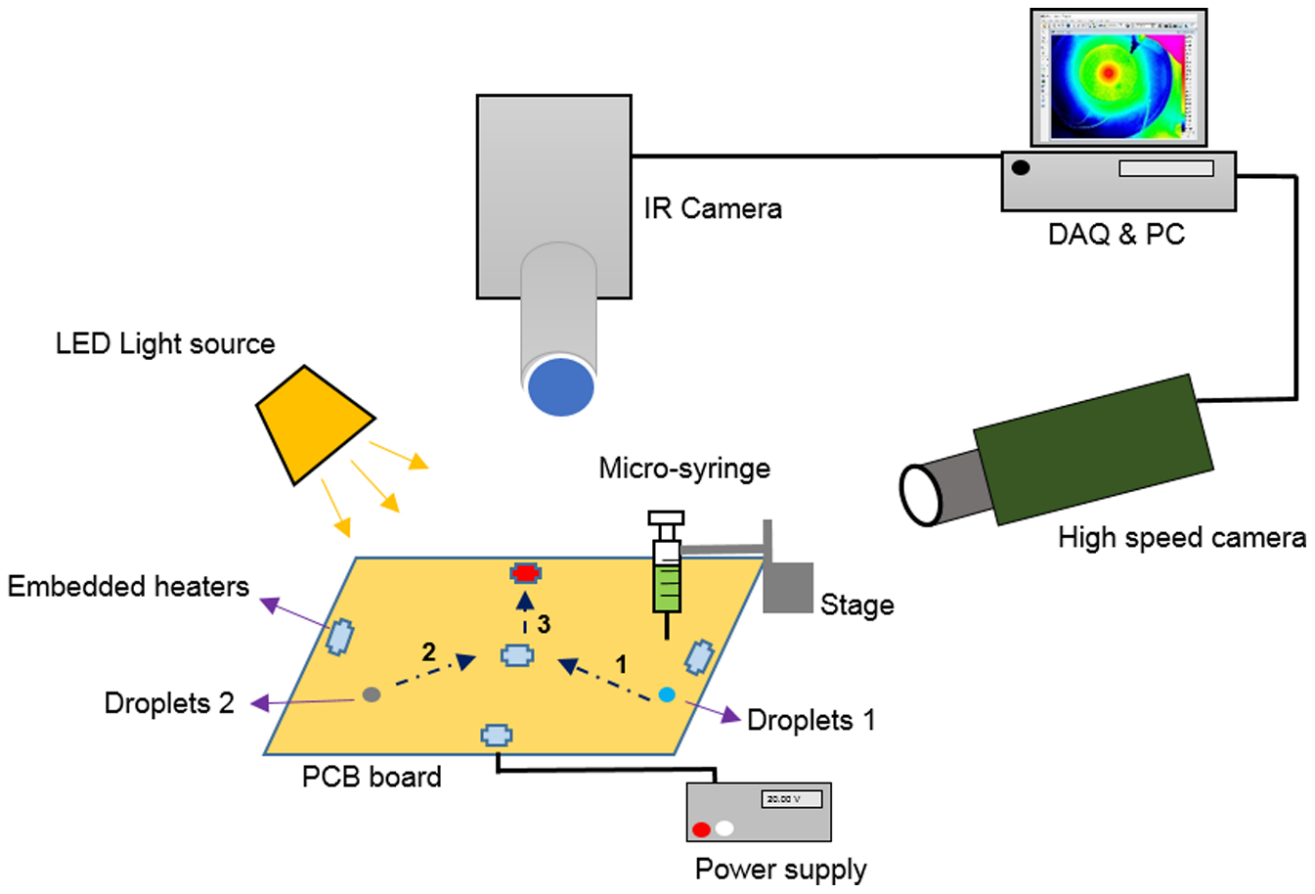
The experimental setup to study the collision of droplets on thin liquid films due to Marangoni effect. The micro-heaters are made out of copper. Light source and cameras are aligned with the droplet migration. An infrared camera is used to monitor the temperature variation of droplets and the carrier liquid. Droplets are produced from a precision needle that is kept at a constant height from the film.

Figure 8 shows the experimental setup. The setup includes a PCB board with embedded heaters, a micropositioning system, electric power supply, imaging acquisition equipment and a droplet generator. Droplets are generated by gently pressing the plunger of a micro-syringe (Terumo). The inner-diameter of the needles are ~0.210–0.108 mm generating droplets with diameter ~2.5–3.6 mm. The syringe is positioned in a stage and kept at a constant height ~1.5 times the droplet diameter over the film. The droplets of size 3 mm are released from the needle tip due to a gravity driven pinch-off. A DC power supply (HC3002, TE) is used to pass electric current through the embedded titanium strips and generate heat due to its electric resistance. The applied voltage is between 20–40 V.

Imaging hardware used in the setup includes a high-speed camera (i-Speed 2, Olympus), 2000 frames per second (fps) and resolution of 576 × 432, a magnification zoom lens (Zoom 7000, Navitar), a digital SLR camera (DS126071, Canon) and a LED light source (LG). The impact parameter and impact velocity are obtained through analysis of the high-speed video images using MATLAB.

In order to track the temperature profile of the film and droplet, an infrared thermal imaging system is used. The IR-camera (SC5000, FLIR) can take images over 380 fps at full resolution of 640 × 512 pixels. The temperature range used in the current experiment is 22–67°C and with an integration time of 1.17 ms. The video images are analyzed using Altair software developed by FLIR systems.

## Supplementary Material

Supplementary Information Supplementary information
