# Supplementary material for: Thermally induced collision of droplets in an immiscible outer fluid
Source: Sci Rep. 2015 May 7;5:9531. doi: 10.1038/srep09531 (PMC5386213; doi:10.1038/srep09531)
Supplement: Supplementary Information [file srep09531-s1.pdf]

Supporting Information

Thermally induced collision of droplets in an immiscible outer fluid

Ashkan Davanlou & Ranganathan Kumar

Mechanical & Aerospace Engineering, University of Central Florida, Orlando, Florida 32816, USA.

Correspondence and requests for materials should be addressed to R.K. (ranganathan.kumar@ucf.edu)

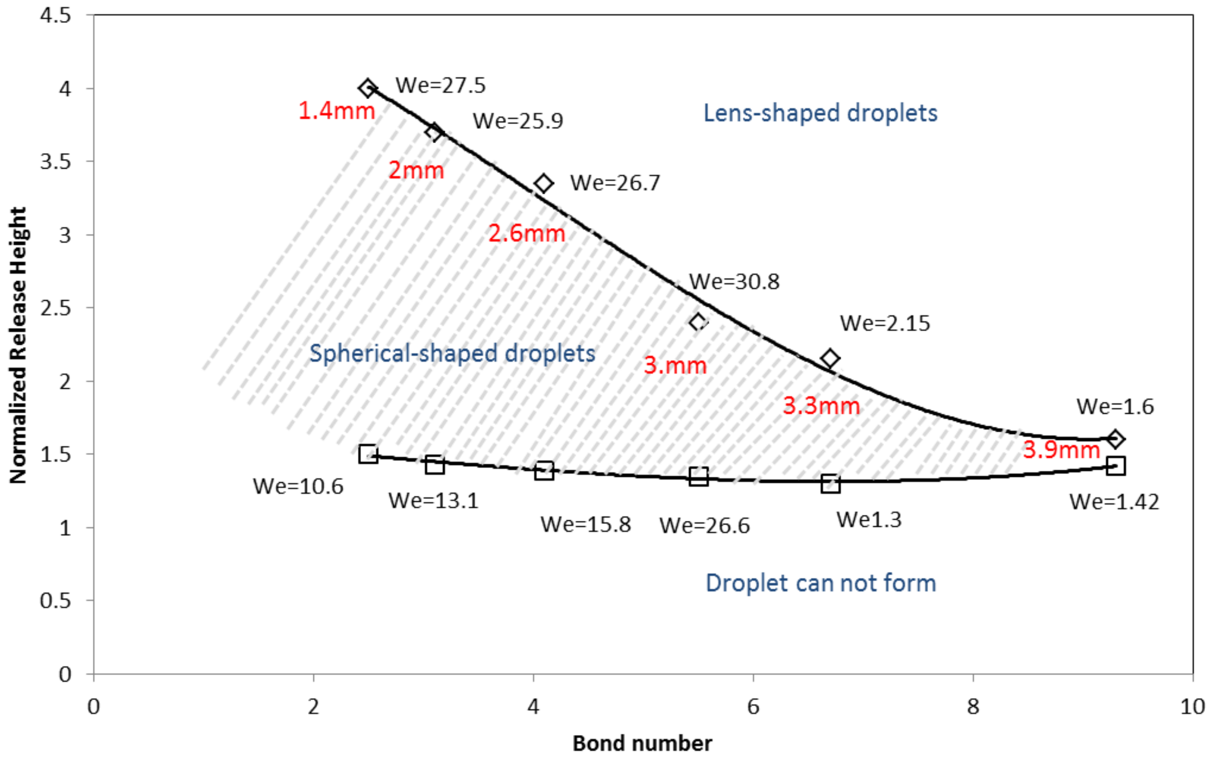

Figure S1. Normalized release height as a function of Bond number ($Bo = \frac{\rho g H^2}{\sigma}$, where H is the releasing height). Droplet diameters are indicated in red. Weber number is calculated as $We = \frac{\rho U^2 D_{drop}}{\sigma}$, where U is the impact speed which is a function of release height given as $U = \sqrt{2gH}$.

Table S1. Physical properties of tested liquids. Some of the properties are taken from ref. [42].

Liquid	ρ [g/cm^3]	ν [centiStoke]	σ [mN/m]	γ [mN/m]
Fluorinert (FC-43)	1.88	2.8	16	-
Water	1	1	71	52
Potassium hydroxide (0.01M)	1	1.05	72.1	-
Phenolphthalein	1.3	2.05	29.5	-
Ethanol	0.79	1.52	22	17.8
Silicon oil 20 cSt	0.95	20	20.6	5.42
Silicon oil 50 cSt	0.96	50	20.8	5.68
Silicon oil 100 cSt	0.966	100	20.9	5.51
